# Supplementary material for: Enzymatic Properties of Recombinant Phospho-Mimetic Photorespiratory Glycolate Oxidases from Arabidopsis thaliana and Zea mays
Source: Plants (Basel). 2019 Dec 24;9(1):27. doi: 10.3390/plants9010027 (PMC7020226; doi:10.3390/plants9010027)
Supplement: Supplementary file 1 [file plants-09-00027-s001.pdf]

Table S1. Primers for site-directed mutagenesis and DNA sequencing.

Primers name	Sequence (5' to 3')
**Site-directed mutagenesis**	
AtGOX1-T4Vf	CATATGGCTAGCGAGATCGTTAACGTTACCGAGTATG
AtGOX1-T4Vr	CATACTCGGTAACGTTAACGATCTCGCTAGCCATATG
AtGOX1-T4Df	GCCATATGGCTAGCGAGATCGATAACGTTACCGAGTATGATG
AtGOX1-T4Dr	CATCATACTCGGTAACGTTATCGATCTCGCTAGCCATATGGC
AtGOX2-T4Vf	TCGCGGATCCATGGAGATCGTTAACGTTACCGAGT
AtGOX2-T4Vr	ACTCGGTAACGTTAACGATCTCCATGGATCCGCGA
AtGOX2-T4Df	TCGCGGATCCATGGAGATCGATAACGTTACCGAGT
AtGOX2-T4Dr	ACTCGGTAACGTTATCGATCTCCATGGATCCGCGA
ZmGO1-T5Vf	GCTAGCGGGGAGATCGTCAATGTCATGGAGT
ZmGO1-T5Vr	ACTCCATGACATTGACGATCTCCCCGCTAGC
ZmGO1-T5Df	TGGCTAGCGGGGAGATCGACAATGTCATGGAGTACC
ZmGO1-T5Dr	GGTACTCCATGACATTGTCGATCTCCCCGCTAGCCA
AtGOX-T158Vf	TGCTCTCACTGTAGACGTCCCAAGGCTAGG
AtGOX-T158Vr	CCTAGCCTTGGGACGTCTACAGTGAGAGCA
AtGOX-T158Df	TGCTCTCACTGTAGACGACCCAAGGCTAGG
AtGOX-T158Dr	CCTAGCCTTGGGTCGTCTACAGTGAGAGCA
ZmGO1-T159Vf	GCTCACCGTCGACGTGCCGCGCCTTGGC
ZmGO1-T159Vr	GCCAAGGCGCGGCACGTCGACGGTGAGC
ZmGO1-T159Df	CGCTCACCGTCGACGACCCGCGCCTTGGCCG
ZmGO1-T159Dr	CGGCCAAGGCGCGGGTCGTCGACGGTGAGCG
AtGOX1-S212Af	ACATCCTTCCAGGCTAAGGTACGGTCAATT
AtGOX1-S212Ar	AATTGACCGTACCTTAGCCTGGAAGGATGT
AtGOX1-S212Df	ACATCCTTCCAGTCTAAGGTACGGTCAATT
AtGOX1-S212Dr	AATTGACCGTACCTTAGACTGGAAGGATGT
AtGOX2-S212Af	GGATATCCTTCCAGGCCAAGGTACGGTCAA
AtGOX2-S212Ar	TTGACCGTACCTTGGCCTGGAAGGATATCC
AtGOX2-S212Df	GGATATCCTTCCAGTCCAAGGTACGGTCAA
AtGOX2-S212Dr	TTGACCGTACCTTGGACTGGAAGGATATCC
ZmGO1-S213Af	TGACGTCTTTCCAGGCCAGGGTGCGGTCGA
ZmGO1-S213Ar	TCGACCGCACCCTGGCCTGGAAAGACGTCA
ZmGO1-S213Df	TGACGTCTTTCCAGTCCAGGGTGCGGTCGA
ZmGO1-S213Dr	TCGACCGCACCCTGGACTGGAAAGACGTCA

AtGOX1-T265Af	GGGCCGAGATGGCTGCTGGGACATAGTC
AtGOX1-T265Ar	GACTATGTCCCAGCAGCCATCTCGGCCC
AtGOX1-T265Df	ACTATGTCCCAGCAGACATCTCGGCCCTTG
AtGOX1-T265Dr	CAAGGGCCGAGATGTCTGCTGGGACATAGT
AtGOX2-T265Af	GATTATGTCCCAGCAGCAATCTCAGCCCTTG
AtGOX2-T265Ar	CAAGGGCTGAGATTGCTGCTGGGACATAATC
AtGOX2-T265Df	CTTGATTATGTCCCAGCAGATATCTCAGCCCTTGAA
AtGOX2-T265Dr	TTCAAGGGCTGAGATATCTGCTGGGACATAATCAAG
ZmGO1-T266A-f	CGCGCTGATGGCCGCCGGCACGT
ZmGO1-T266A-r	ACGTGCCGGCGGCCATCAGCGCG
ZmGO1-T266D-f	CTACGTGCCGGCGGACATCAGCGCGCTG
ZmGO1-T266D-r	CAGCGCGCTGATGTCCGCCGGCACGTAG
**DNA sequencing**	
T7	TAATACGACTCACTATA
T7term	GCTAGTTATTGCTCAGCGG

```
AtGOX1          -MEITNVTEYDAIAKQKLPKMVYDYYASGAEDQWTLQENRNAFARILFRPRILIDVSKID 59
AtGOX2          -MEITNVTEYDAIAKAKLPKMVYDYYASGAEDQWTLQENRNAFARILFRPRILIDVNKID 59
AtGOX3          -MEITNVMEYEKIAKEKLPKMVYDYYASGAEDQWTLQENRNAFSRILFRPRILIDVSKID 59
AtHAOX1         MDQIVNVDEFQELAKQALPKMYYDFYNGGAEDQHTLNENVQAFRRIMFRPRVLVDVSNID 60
AtHAOX2         MDQIVNVDEFQELAKQALPKMYYDFYNGGAEDQHTLNENVQAFRRIMFRPRVLVDVSKID 60
                  :*.** *:: :**  **** **:* .***** **:** :** **:****:*:**.:**

AtGOX1          MTTTVLGFKISMPIMVAPTAMQKMAHPDGEYATARAASAAGTIMTLSSWATSSVEEVAST 119
AtGOX2          MATTVLGFKISMPIMVAPTAFQKMAHPDGEYATARAASAAGTIMTLSSWATSSVEEVAST 119
AtGOX3          VSTTVLGFNISMPIMIAPTAMQKMAHPDGELATARATSAAGTIMTLSSWATCSVEEVAST 119
AtHAOX1         MSTSMLGYPISAPIMIAPTAMHKLAHPKGEIATAKAAAACNTIMIVSFMSTCTIEEVASS 120
AtHAOX2         MSTKILGYPISAPIMIAPTGNHKLAHPEGETATAKAAAACNTIMIVSYMSSCTFEEIASS 120
                ::*.:**: ** ***:***. :*:***.** ***:*::*..*** :*  ::.:.**:**:

                                                         Loop4  α-Helix4
AtGOX1          GPGIRFFQLYVYKNRNVVEQLVRRAERAGFKAIALTVDTPRLGRRESDIKNRFTLPPNLT 179
AtGOX2          GPGIRFFQLYVYKNRKVVEQLVRRAEKAGFKAIALTVDTPRLGRRESDIKNRFTLPPNLT 179
AtGOX3          GPGIRFFQLYVYKDRNVVIQLVKRAEEAGFKAIALTVDTPRLGRRESDIKNRFALPRGLT 179
AtHAOX1         CNAVRFLQIYVYKRRDVTAQIVKRAEKAGFKAIVLTVDVPRLGRREADIKNKMISP---Q 177
AtHAOX2         CNAVRFLQIYVYKRRDITAQVVKRAEKAGFKAIVLTVDVPRLGRREADIKNKMISP---Q 177
                  .:**:*:**** *.:. *:*:***.******.****.*******:****::   *

AtGOX1          LKNFEGLDLGKMDEANDSGLASYVAGQIDRTLSWKDVQWLQTITKLPILVKGVLTGEDAR 239
AtGOX2          LKNFEGLDLGKMDEANDSGLASYVAGQIDRTLSWKDIQWLQTITNMPILVKGVLTGEDAR 239
AtGOX3          LKNFEGLDLGKIDKTNDSGLASYVAGQVDQSLSWKDIKWLQSITSLPILVKGVITAEDAR 239
AtHAOX1         LKNFEGLVSTEVRPNEGSGVEAFASSAFDASLSWKDIEWLRSITKLPILVKGLLTREDAL 237
AtHAOX2         LKNFEGLFSTEVRPSKGSGVQAFASRAFDASFSWKDIEWLRSITELPILVKGILTREDAL 237
                *******    ::    :.**: ::.:   .* ::****::**::**.:******::* ***

                                  Loop6           α–Helix6
AtGOX1          IAIQAGAAGIIVSNHGARQLDYVPATISALEEVVKATQGRIPVFLDGGVRRGTDVFKALA 299
AtGOX2          IAIQAGAAGIIVSNHGARQLDYVPATISALEEVVKATQGRVPVFLDGGVRRGTDVFKALA 299
AtGOX3          IAVEYGAAGIIVSNHGARQLDYVPATIVALEEVVKAVEGRIPVFLDGGVRRGTDVFKALA 299
AtHAOX1         KAVEAGVDGIVVSNHGARQLDYSPATITVLEEVVHAVKGRIPVLLDGGVRRGTDVFKALA 297
AtHAOX2         KAVEAGVDGIIVSNHGGRQLDYSPATITVLEEVVQVVRGRIPVLLDGGVRRGTDVFKALA 297
                 *:: *. **:*****.***** ****  .*****:...**:**:****************

AtGOX1          LGASGIFIGRPVVFSLAAEGEAGVRKVLQMLRDEFELTMALSGCRSLKEISRNHITTEWD 359
AtGOX2          LGASGIFIGRPVVFALAAEGEAGVKKVLQMLRDEFELTMALSGCRSLSEITRNHIVTEWD 359
AtGOX3          LGASGVFVGRPSLFSLAADGEAGVRKMLQMLRDEFELTMALSGCRSLREISRTHIKTDWD 359
AtHAOX1         LGAQAVLIGRPIVYGLAAKGEDGVKKVIDMLKNEFEITMALSGCPTIDDVTRNHVRTENE 357
AtHAOX2         LGAQAVLIGRPIIYGLAAKGEDGVKKVIDMLKNEFEITMALSGCPTIDDITRNHVRTENE 357
                ***..:::*** ::.***.** **:*:::**::***:******* :: :::*.*: *: :

AtGOX1          TPRPS-ARL 367
AtGOX2          TPRHL-PRL 367
AtGOX3          TPHYLSAKL 368
AtHAOX1         RIKSML--- 363
AtHAOX2         RLHSML--- 363
                  :
```

Figure S1. *At*GOX1, *At*GOX2, *At*GOX3, *At*HAOX1 and *At*HAOX2 protein sequence alignments showing the conservation of phosphorylated residues and other important residues and structures

Sequence alignment was carried out using Clustal Omega software (https://www.ebi.ac.uk/Tools/msa/clustalo/). Phosphorylated amino acids T4, T155, T158, S212, T265 and T355 are highlighted in red. When the phosphorylated amino acid is replaced by a valine in *At*HAOX1 and *At*HAOX2 proteins, they are highlighted in green. Loop4 and α–helix4 are highlighted in yellow and loop6 and α–helix6 are highlighted in blue. Residues

involved in FMN binding are marked by stars [27]. Residues involved in the catalytic site are marked by circles [28,29]. UniProtKB: Q9LRR9 (*At*GOX1), F4JFV6 (*At*GOX2), O49506 (*At*GOX3), Q9LJH5 (*At*HAOX1) and Q24JJ8 (*At*HAOX2).

```
Aerococcus             ------------mnnndieynapseikyidvvntydleee----askvvphggfnyiaga  44
Lactococcus            mhlsstdvnfiknrrtkmtyktstenkaleivnvkslegkvkqsmeaagnkggfgyirgg  60
Homo_HAOX2             ----------------------------mslvcltdfqah----areqlskstrdfiegg  28
Chlamydomonas          -------------------------madlsflnleeveee----akkvmpkmafdyystg  31
Nostoc                 ------------------------mtaisspinlfeyeql----akthlsqmafdyyisg  32
Homo_GOX1              --------------------------mlprlicindyeqh----aksvlpksiydyyrsg  30
Arabidopsis_HAOX1      ---------------------------mdqivnvdefqel----akqalpkmyydfyngg  29
Arabidopsis_HAOX2      ---------------------------mdqivnvdefqel----akqalpkmyydfyngg  29
Arabidopsis_GOX3       ----------------------------meitnvmeyeki----akeklpkmvydyyasg  28
Zea                    ---------------------------mgeitnvmeyqai----akqklpkmaydyyasg  29
Spinacia               ----------------------------meitnvneyeai----akqklpkmvydyyasg  28
Arabidopsis_GOX1       ----------------------------meitnvteydai----akqklpkmvydyyasg  28
Arabidopsis_GOX2       ----------------------------meitnvteydai----akaklpkmvydyyasg  28
Brassica               ----------------------------meitnvteyeai----akeklpkmvydyyasg  28
Vitis                  ----------------------------meitnvteyeai----akqklpkmvfdyyasg  28
Nicotiana              ---------------------------meevtnvmeyeai----akkklpkmvfdyyasg  29
Populus                ----------------------------meitnvteyeai----akqklpkmafdyyasg  28
                                                          . :            :   .:   .

Aerococcus             sgdewtkrandrawkhkllyprlaqdveapdtsteilghkikapfimapiaahglahttk  104
Lactococcus            sedewtlnentsafnkkqimprvlrgidsadlstslfgiklktpiiqapvaaqglahaeg  120
Homo_HAOX2             addsitrddniaafkrirlrprylrdvsevdtrttiqgeeisapiciaptgfhclvwpdg  88
Chlamydomonas          sdtcytvgenrscfsrylllprmlrnvsrvdtshelfgirssmpvwvapmamhglahpgr  91
Nostoc                 agdeitlqenravferiklrprmlvdvsqinlttsvlgqplqlplliapmafqclahteg  92
Homo_GOX1              andeetladniaafsrwklyprmlrnvaetdlstsvlgqrvsmpicvgatamqrmahvdg  90
Arabidopsis_HAOX1      aedqhtlnenvqafrrimfrprvlvdvsnidmstsmlgypisapimiaptamhklahpkg  89
Arabidopsis_HAOX2      aedqhtlnenvqafrrimfrprvlvdvskidmstkilgypisapimiaptgnhklahpeg  89
Arabidopsis_GOX3       aedqwtlqenrnafsrilfrprilidvskidvsttvlgfnismpimiaptamqkmahpdg  88
Zea                    aedewtlqenreafsrilfrprilidvskidmtttvlgfkismpimvaptamqkmahpdg  89
Spinacia               aedqwtlaenrnafsrilfrprilidvtnidmtttilgfkismpimiaptamqkmahpeg  88
Arabidopsis_GOX1       aedqwtlqenrnafarilfrprilidvskidmtttvlgfkismpimvaptamqkmahpdg  88
Arabidopsis_GOX2       aedqwtlqenrnafarilfrprilidvnkidmattvlgfkismpimvaptafqkmahpdg  88
Brassica               aedqwtlqenrnafarilfrprilidvskidmtttvlgfkismpimvaptamqkmahpeg  88
Vitis                  aedqwtlyqnrhafsqilfrprilidvskidmtttvlgfkismpimiaptamqkmahpeg  88
Nicotiana              aedqwtlaenrnafsrilfrprilidvskidmsttvlgfkismpimiaptamqkmahpeg  89
Populus                aedqwtlaenrnafsrilfrprilidvskidiattvlgfkismpimiaptafqkmahpeg  88
                       :    *   *   : :  : **   .:   :    : *   . *.  .  . : :.

Aerococcus             eagtaravsefgtimsisaysgatfeeisegln---ggprwfqiymakddqqnrdildea  161
Lactococcus            evatakamaevgsifsistygstsvedaakaap---dapqffqlymskddkfnefllkka  177
Homo_HAOX2             emstaraaqaagicyitstfascslediviaap---eglrwfqlyvhpdlqlnkqliqrv  145
Chlamydomonas          evatcraaaaagvpftfstvatsslqeiqetgh----dnrifqlyvirnrevvrrwvtea  147
Nostoc                 elatamaaasagtgmvlstlstksleevaevgskfspslqwfqlyihkdrgltralvera  152
Homo_GOX1              elatvracqslgtgmmlsswatssieevaeagp---ealrwlqlyiykdrevtkklvrqa  147
Arabidopsis_HAOX1      eiatakaaaacntimivsfmstctieevasscn----avrflqiyvykrrdvtaqivkra  145
Arabidopsis_HAOX2      etatakaaaacntimivsymssctfeeiasscn----avrflqiyvykrrditaqvvkra  145
Arabidopsis_GOX3       elataratsaagtimtlsswatcsveevastgp----girffqlyvykdrnvviqlvkra  144
Zea                    enataraaaaagtimtlsswatssveevastgp----girffqlyvykdrkvveqlvrra  145
Spinacia               eyataraasaagtimtlsswatssveevastgp----girffqlyvykdrnvvaqlvrra  144
Arabidopsis_GOX1       eyataraasaagtimtlsswatssveevastgp----girffqlyvyknrnvveqlvrra  144
Arabidopsis_GOX2       eyataraasaagtimtlsswatssveevastgp----girffqlyvyknrkvveqlvrra  144
Brassica               eyataraasaagtimtlsswatssveevastgp----girffqlyvyknrkvveqlvrra  144
Vitis                  eyataraasatgtimtlsswatssveevastgp----girffqlyvykdrhvvaqlvrra  144
Nicotiana              eyataraasaagtimtlsswatssveevastgp----girffqlyvykdrnvvaqlvrra  145
Populus                eyataraasaagtimtlsswatssveevastgp----girffqlyvykdrnvvaqlvrra  144
                       * .*  *    .     *  .  :.::            : :*:*:          : ..

Aerococcus             ksdgataiiltadstvsgnrdrdvknkfvypfgmpi--vqrylrg------taegmslnn  213
Lactococcus            vsagvkaiiltadstlggyreedivnhfqfpfpmpn--laafses------dgtgkgise  229
Homo_HAOX2             eslgfkalvitldtpvcgnrrhdirnqlrr--nltltdlqs----------pkkgnaipy  193
Chlamydomonas          esrgfkalmvtvdaqrlgnreadarnkftlppglalrnleylssas-tardsqdgsglmk  206
Nostoc                 yaagykalcltvdapvlgqrerdrrnefvlppglhlanlttisglni--phapgesglft  210
Homo_GOX1              ekmgykaifvtvdtpylgnrlddvrnrfklppqlrmknfetstlsfspeenfgddsglaa  207
Arabidopsis_HAOX1      ekagfkaivltvdvprlgrreadiknkmispq---lknfeglvste---vrpnegsgvea  199
Arabidopsis_HAOX2      ekagfkaivltvdvprlgrreadiknkmispq---lknfeglfste---vrpskgsgvqa  199
Arabidopsis_GOX3       eeagfkaialtvdtprlgrresdiknrfalprgltlknfegldlgk---idktndsglas  201
Zea                    eragfkaialtvdtprlgrreadiknrfvlpphltlknfegldlgk---mdqaadsglas  202
```

```
Spinacia                eragfkaialtvdtprlgrreadiknrfvlppfltlknfegidlgk---mdkandsglss  201
Arabidopsis_GOX1        eragfkaialtvdtprlgrresdiknrftlppnltlknfegldlgk---mdeandsglas  201
Arabidopsis_GOX2        ekagfkaialtvdtprlgrresdiknrftlppnltlknfegldlgk---mdeandsglas  201
Brassica                ekagfkaialtvdtprlgrresdiknrftlppnltlknfegldlgk---mdeandsglas  201
Vitis                   eragfkaialtvdtprlgrreadiknrftlppfltlknfegldlgk---mdkaddsglas  201
Nicotiana               eragfkaialtvdtprlgrreadiknrfvlppfltlknfegldlgk---mdqasdsglas  202
Populus                 eragfkaialtvdtprlgrresdiknrftlppfltlknfegldlgk---mdkandsglas  201
                           * .*: :* *    * *  *  *.:          .                 .:

Aerococcus              iyg-askqkisprdieeiaghsglpvfvkgiqhpedadmaikrgasgiwvsnhgarqlye  272
Lactococcus             iya-aakqglvlediqkikkitnlpvivkgvqspidaddainagadgiwvsnhggrqldg  288
Homo_HAOX2              fqmtpistslcwndlswfqsitrlpiilkgiltkedaelavkhnvqgiivsnhggrqlde  253
Chlamydomonas           lftsevddsltwefipwlrgvtklpiivkgllspadaelavqygvdgivvsnhggrqldy  266
Nostoc                  yfaqqlnpaltwddlewlqslsplplvlkgilrgddaaraveygakaivvsnhggrqldg  270
Homo_GOX1               yvakaidpsiswedikwlrrltslpivakgilrgddareavkhglngilvsnhgarqldg  267
Arabidopsis_HAOX1       fassafdaslswkdiewlrsitklpilvkglltredalkaveagvdgivvsnhgarqldy  259
Arabidopsis_HAOX2       fasrafdasfswkdiewlrsitelpilvkgiltredalkaveagvdgiivsnhggrqldy  259
Arabidopsis_GOX3        yvagqvdqslswkdikwlqsitslpilvkgvitaedariaveygaagiivsnhgarqldy  261
Zea                     yvagqvdrtlswkdvkwlqtittlpilvkgvltaedtrlavangaagiivsnhgarqldy  262
Spinacia                yvagqidrslswkdvawlqtitslpilvkgvitaedarlavqhgaagiivsnhgarqldy  261
Arabidopsis_GOX1        yvagqidrtlswkdvqwlqtitklpilvkgvltgedariaiqagaagiivsnhgarqldy  261
Arabidopsis_GOX2        yvagqidrtlswkdiqwlqtitnmpilvkgvltgedariaiqagaagiivsnhgarqldy  261
Brassica                yvagqidrtlswkdvqwlqtitsmpilvkgvltgedariaiqagaagiivsnhgarqldy  261
Vitis                   yvagqidrtlswkdvkwlqtitnlpilvkgvltaedtrlaiqagaagiivsnhgarqldy  261
Nicotiana               yvagqidrtlswkdvqwlqtitslpilvkgvltaedarlavqagaagiivsnhgarqldy  262
Populus                 yvagqidrtlswkdvqwlqtitrlpilvkgvltaedarlsiqagaagiivsnhgarqldy  261
                              .  :    :  :   : :*:. **:    *:  ::  .  .* *****.***

Aerococcus              apgsfdtlpaiaervnk---rvpivfdsgvrrgehvakalasgadvvalgrpvlfglalg  329
Lactococcus             gpasidvlpliaksvnh---rvpivfdsgvrrgehvfkalaqgadvvavgrpvlyglnlg  345
Homo_HAOX2              vlasidaltevvaavkg---kievyldggvrtgndvlkalalgakciflgrpilwglack  310
Chlamydomonas           apsglhmlpavvaavrgcgssipvlvdggvrrgtdvikalalgasgvllgrpvlyglavg  326
Nostoc                  aiasldalpeivaavng---kaevlldggirrgtdiikalaigaqavligrpvlwglavg  327
Homo_GOX1               vpatidvlpeiveaveg---kvevfldggvrkgtdvlkalalgakavfvgrpivwglafq  324
Arabidopsis_HAOX1       spatitvleevvhavkg---ripvlldggvrrgtdvfkalalgaqavligrpivyglaak  316
Arabidopsis_HAOX2       spatitvleevvqvvrg---ripvlldggvrrgtdvfkalalgaqavligrpiiyglaak  316
Arabidopsis_GOX3        vpativaleevvkaveg---ripvfldggvrrgtdvfkalalgasgvfvgrpslfslaad  318
Zea                     vpatisaleevvkaarg---qlpvfvdggvrrgtdvfkalalgaagvfvgrpvvfslaaa  319
Spinacia                vpatimaleevvkaaqg---ripvfldggvrrgtdvfkalalgaagvfigrpvvfslaae  318
Arabidopsis_GOX1        vpatisaleevvkatqg---ripvfldggvrrgtdvfkalalgasgifigrpvvfslaae  318
Arabidopsis_GOX2        vpatisaleevvkatqg---rvpvfldggvrrgtdvfkalalgasgifigrpvvfalaae  318
Brassica                vpatisaleevvkatqg---rvpvfldggvrrgtdvfkalalgasgifigrpvvfslaae  318
Vitis                   vpatimaleevvkaaqg---rvpvfldggvrrgtdvfkalalgasgifigrpvvfslaae  318
Nicotiana               vpstimaleevvkaaqg---ripvfldggvrrgtdvfkalalgasgifigrpvvfslaae  319
Populus                 vpstimaleevvkaaqg---rvpvfldggvrrgtdvfkalalgasgifigrpvvfslase  318
                          . :  *  :.  ..       : .*.*:* * .: **** **  : :*** ::.*

Aerococcus              gwqgaysvldyfqkdltrvmqltgsqnvedlkgldlfdnpygyey-----------      374
Lactococcus             gakgvqsvfehlnkelsitmqlagtknieeikhtslid------------------      383
Homo_HAOX2              gehgvkevlniltnefhtsmaltgcrsvaeinrnlvqfsrl---------------      351
Chlamydomonas           gqagvervlqllrseielsmalagcssvqqigpqlllpapsagpappmpaaqlckl      382
Nostoc                  gqagvshvisllqkelnvamaligcsqlqdidtsflhl------------------      365
Homo_GOX1               gekgvqdvleilkeefrlamalsgcqnvkvidktlvrknplavski----------      370
Arabidopsis_HAOX1       gedgvkkvidmlknefeitmalsgcptiddvtrnhvrteneriksml---------      363
Arabidopsis_HAOX2       gedgvkkvidmlknefeitmalsgcptidditrnhvrtenerlhsml---------      363
Arabidopsis_GOX3        geagvrkmlqmlrdefeltmalsgcrslreisrthiktdwdtphylsakl------      368
Zea                     geagvsnvlrmlrdefeltmalsgctslaeitrkhiitesdklsaipsrl------      369
Spinacia                geagvkkvlqmmrdefeltmalsgcrslkeisrshiaadwdgpssrav--arl---      369
Arabidopsis_GOX1        geagvrkvlqmlrdefeltmalsgcrslkeisrnhittewdtprpsarl-------      367
Arabidopsis_GOX2        geagvkkvlqmlrdefeltmalsgcrslseitrnhivtewdtprhlprl-------      367
Brassica                geagvrkvlqmlrdefeltmalsgcrslseitrnhiitewetprhlprl-------      367
Vitis                   geagvrkvlqmlreefeltmalsgcrslkeitrdhivtewevphpgsrplprl---      371
Nicotiana               geagikkvlqmlrdefeltmalsgcrslneitrnhivtewdapraa-lpaprl---      371
Populus                 geagvrkvlqmlreefeltmalsgcrslkeitrnhivtdwdhpral-lapk-l---      369
                        *  *   ::  : .::   * * *   :  :    :
```

Figure S2. Amino acid sequence alignment of *Lactococcus lactis*, *Aerococcus viridians*, *Nostoc* and *Chlamydomonas reinhardtii* lactate oxidases with glycolate oxidases and hydroxy-acid oxidases of human and plants.

Sequence alignment was carried out using Clustal Omega software (https://www.ebi.ac.uk/Tools/msa/clustalo/). Phosphorylated amino acids T4, T155, T158, S212, T265 and T355 from *Arabidopsis thaliana* and their equivalents in other proteins are highlighted in red. Amino acids in yellow were presumed to be involved in substrate binding (Hackenberg et al, 2011). UniProtKB: Lactate oxidase: Q44467 (*Aerococcus viridians* IF012219), Q9CG58 (*Lactococcus lactis* Il1403), Q8Z0C8 (*Nostoc sp. PCC 7120*), A8IEL8 (*Chlamydomonas reinhardtii*) ; HAOX : Q9LJH5 (*Arabidopsis thaliana,* HAOX1), Q24JJ8 (*Arabidopsis thaliana,* HAOX2), Q9NYQ3 (Homo sapiens, HAOX2) ; GOX : C0P702 (*Zea mays*), Q9LRR9 (*Arabidopsis thaliana*, *At*GOX1), F4JFV6 (*Arabidopsis thaliana*, *At*GOX2), O49506 (*Arabidopsis thaliana*, *At*GOX3), Q3L1H0 (*Brassica napus*), A5B1R1 (*Vitis vinifera*), E1AXT8 (*Nicotiana benthamiana*), A0A4U5NR44 (*Populus alba*), P05414 (*Spinacia oleracea*), and Q9UJM8 (Homo sapiens, GOX1).

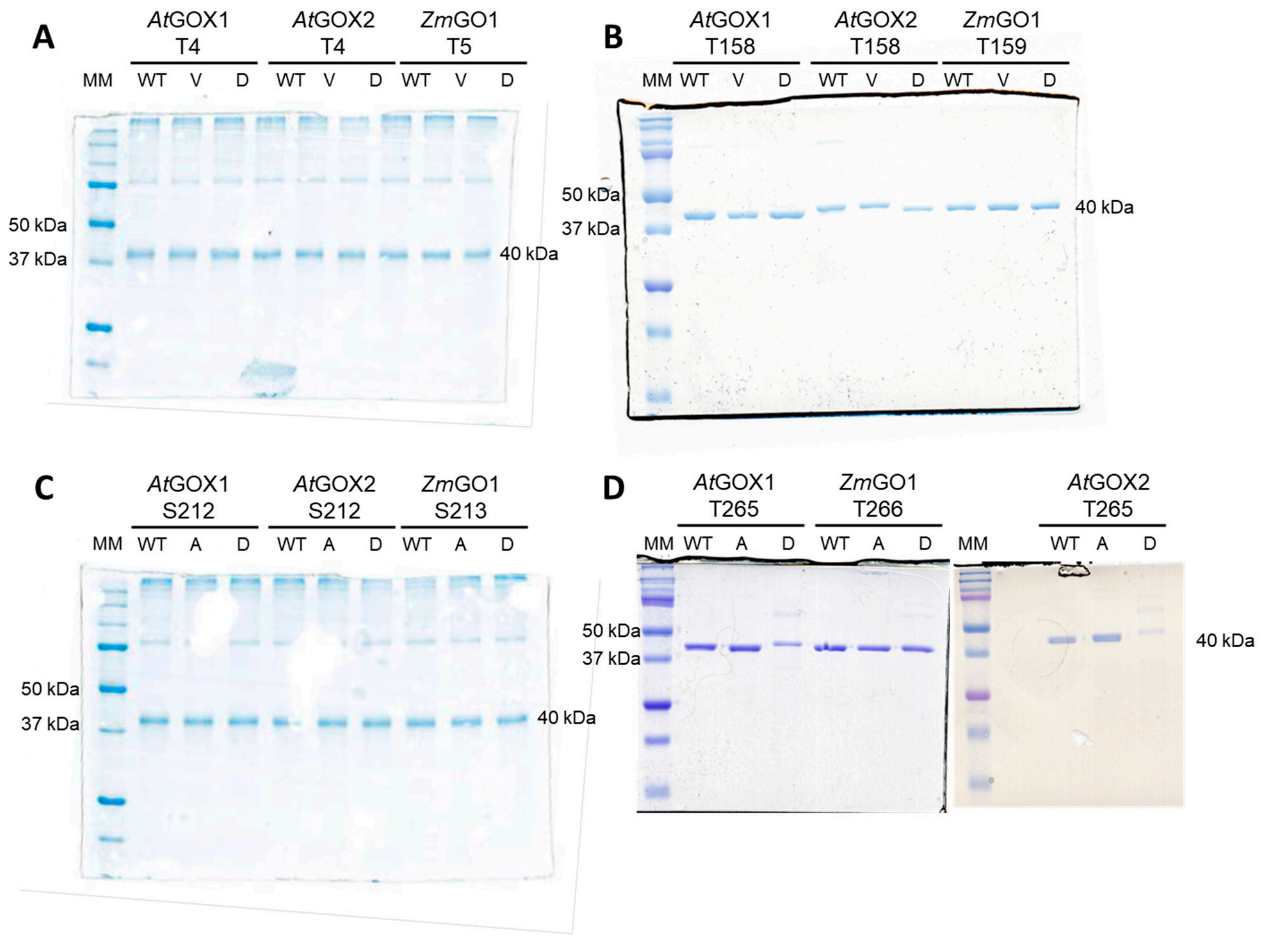

Figure S3. Purified recombinant His-tag proteins of wild-type *At*GOX1, *At*GOX2 and *Zm*GO1 and their phosphosite mutated forms on Coomassie-stained SDS-PAGE gels. Two micrograms of desalted purified GOX proteins were separated by SDS-PAGE with 10% acrylamide and stained with Coomassie Blue R-250. Purified recombinant proteins of wild-type *At*GOX1, *At*GOX2 and *Zm*GO1 and (A) *At*GOX1/2 T4V/D and *Zm*GO1 T5V/D, (B) *At*GOX1/2 T158V/D and *Zm*GO1 T159V/D, (C) *At*GOX1/2 S212A/D and *Zm*GO1 S213A/D, and (D) *At*GOX1/2 T265A/D and *Zm*GO1 T266A/D. Molecular mass markers (MM) are shown with the molecular masses of 50 kDa and 37 kDa indicated. Each GOX recombinant protein was estimated to be ~40 kDa.
